# Digital health literacy and well-being of university students in Austria during the pandemic

**DOI:** 10.1093/eurpub/ckac129.710

**Published:** 2022-10-25

**Authors:** F Reitegger, M Wright, B Gasteiger-Klicpera

**Affiliations:** 1 Institute of Education Research &Teacher Education, University of Graz, Graz, Austria; 2 Research Center for Inclusive Education, University of Graz, Graz, Austria

## Abstract

**Background:**

Previous findings suggest that depressive and anxiety-related symptoms have doubled among students since the beginning of the pandemic. Digital health literacy can act as a protective resource to strengthen well-being.

**Objectives:**

This paper analyzes the relationship between digital health literacy, socioeconomic status and well-being and future-anxiety among students in Austria.

**Methods:**

480 students from Austrian higher education institutions were surveyed via online questionnaire during the second wave of the Corona pandemic. Sociodemographic data, students’ self-assessments of well-being, fears regarding future development and perspectives, and digital health literacy were collected. Variance and regression analyses were used for the evaluation.

**Results:**

About 50% of the students reported low scores in well-being and distinct fears about the future. A higher socioeconomic status correlated with higher well-being as well as lower fears about the future.Regarding digital health literacy, the ability to assess the relevance of information showed the highest correlation with well-being.

**Conclusions:**

Individual factors such as gender or the study-program are relevant for the interaction between well-being and digital health literacy. The assessment of the relevance of information and its connection with one's own life reality seems to be important factors in promoting well-being.

